# Endodontic diagnostics training in undergraduate dental education: An observational pilot study on AI‐driven virtual patient e‐learning

**DOI:** 10.1111/iej.14277

**Published:** 2025-07-22

**Authors:** Marian Prinz, Edgar Schäfer, Sebastian Bürklein, David Donnermeyer

**Affiliations:** ^1^ Department of Periodontology and Operative Dentistry University of Münster Münster Germany; ^2^ Central Interdisciplinary Ambulance in the School of Dentistry University of Münster Münster Germany; ^3^ Department of Restorative, Preventive and Pediatric Dentistry, School of Dental Medicine University of Bern Bern Switzerland

**Keywords:** artificial intelligence (AI), dental education, e‐learning, endodontic diagnostics, generative pre‐trained transformer (GPT), virtual patient

## Abstract

**Aim:**

To develop and evaluate an e‐learning tool utilizing a generative pre‐trained transformer (GPT), a form of artificial intelligence (AI), to allow for realistic conversation on virtual patients when undergoing training on how to diagnose diseases of endodontic origin, and to evaluate improvements in self‐perceived skills.

**Methodology:**

A web app consisting of three components [website for user access, database server with patient case information, GPT‐4‐turbo model (OpenAI)] was designed to serve as the e‐learning platform. Undergraduate students from 4th and 5th year at the dental school of the University of Münster, Germany, were asked to solve eight cases of virtual patients presenting with pain from endodontic or periodontal origin. Before, a questionnaire applying a 5‐point Likert scale served to evaluate the current self‐perceived state regarding the education, experience and skills in endodontic diagnostics and emergency treatment. After a 3‐month timeframe of working with the programme individually, the students were asked to answer a second questionnaire which focused on their experience and self‐perceived skills improvement after using the training software.

**Results:**

Ninety‐two students participated in the first questionnaire and 72 students finished the second questionnaire, resulting in a drop‐out rate of 21.7%. Students in the 5th‐year reported more experience in dealing with emergency patients. Initially, both cohorts mainly did not feel confident to perform endodontic diagnostics independently. The evaluation of confidence to perform endodontic diagnostics independently, both by 4th‐ and 5th‐year students, seemed improved after the training on the e‐learning tool. The tool was recommended to be available for endodontic education by 72.2% of the students, who strongly agreed to such a statement. 76.4% of the participants strongly agreed to recommend the use of the tool to other students.

**Conclusions:**

AI‐based interactive e‐learning programmes allowing for complex conversational patient encounters present a possibility to improve diagnostic and interactive skills of undergraduate students.

## INTRODUCTION

Endodontic therapy presents a challenging procedure in clinical dentistry and is perceived as highly complex and stress inducing by dental students (Friedlander et al., 2023). Along with the procedural challenges of root canal therapy, the perception of enhanced complexity arises from the demanding diagnostics of pulpal and periapical diseases to ensure adequate patient care. Particularly in cases of endodontic emergencies, excellent diagnostic skills and confident decision‐making are crucial to perform suitable treatment alleviating pain promptly and preventing further complications (Abbott, 2022).

Highlighting the importance of acquiring clinical and interpersonal skills and the achievement of self‐efficacy in dental school graduates, the ‘Undergraduate Curriculum Guidelines for Endodontology’ (Baaij et al., 2024), serve as a reference for dental schools and regulatory bodies. Since guidelines must allow for adaptation based on the individual resources and local regulations in dental schools, educational approaches may differ significantly. Particularly in endodontic diagnostics and emergency care, some universities rely solely on lectures and tutorials, dedicating on average just 4 h to this subject (Al Raisi et al., 2019; Mergoni et al., 2022), while others also demand for internships in emergency clinic rotations (Tiwana et al., 2007). Still, with the given resources, there might be a discrepancy between university teaching and the requirements to sufficiently prepare students for their future clinical responsibilities (Anderson et al., 2015), though there is widespread consensus that students should gain sufficient experience in emergency patient care (Baaij et al., 2024). Due to limited resources of clinical chairside teaching, the curriculum guidelines of the European Society of Endodontology (ESE) further recognize simulation exercises as a viable alternative for enriching students' practical skills (Baaij et al., 2024). Besides serving as a substitute for chairside education, simulations could also bridge the gap between classroom learning and first patient encounters linking theoretical knowledge and clinical proficiency (Weller, 2004). Even if urgent care rotations are already part of a curriculum, all students could benefit from simulations, as they on the one hand boost self‐efficacy (McKenzie et al., 2019) and on the other hand may highlight potential knowledge gaps, students can independently work on before being exposed to real patient encounters. This is valuable since students may feel insecure about diagnosing patients independently (Luz et al., 2019).

Several ideas have been proposed to enhance clinical education in endodontics and general dental education, respectively, in the past. Technological advancement in computer technology and artificial intelligence has opened doors for new opportunities, and suggestions have included multimedia patient simulation (Littlefield et al., 2003), a diagnostic training software (Al‐Madi et al., 2020), student role play (Wen et al., 2020) or simulated and virtual patient encounters (Sanchez et al., 2019) to highlight a few.

Among these, the use of simulated patients stands out as the most realistic case‐based learning method. These training scenarios, however, are difficult to implement and obtain. In contrast, virtual patient simulations present a scalable, cost‐effective and flexible alternative, reducing the financial and logistical complexities associated with employing and educating standardized patient actors. Moreover, virtual patient simulations have the potential to ensure that every student encounters a comprehensive range of diagnoses throughout their clinical training, liberating them from the unpredictability of patient distribution of traditional clinical rotations. Therefore, their use is already widely accepted among dental schools (Cederberg et al., 2012) and students perceive them well (Seifert et al., 2019).

The variety of different implementations for virtual patient programmes is extensive and has shifted over time to create more realistic simulations. Initial designs were linear and passive, resembled by paper cases and evolved into more dynamic formats, where the narrative unfolded step‐by‐step, often referred to as the ‘string of pearls’ approach. In these setups, learners could not influence the case outcomes. This changed with the introduction of branched, decision tree approaches, which allowed learners to make choices at certain waypoints during the case, thereby influencing its progression (Zary et al., 2006). Over time, even greater advancements in interactivity were achieved by incorporating AI chatbot tools and thereby enhancing user engagement (Pereira et al., 2023). Suárez et al. (2022) as well as Co et al. (2022) based their approach on the tool dialogflow (Alphabet Inc., Mountinview, California, USA) to enable a less constrained interaction with the virtual patient. Kim et al. (2023) adopted a more complex approach by developing a conformer‐based AI patient model from the ground up to facilitate virtual patient encounters. However, as this research represents foundational work, it appears to have not yet been applied in practical student training.

In recent years, the introduction of Large Language Models (LLMs) and Generative Pre‐trained Transformer (GPT) models as a subgroup, popularized by ChatGPT (OpenAI, San Francisco, CA, USA), has presented significant opportunities for virtual patient simulations. Pretrained on vast amounts of readily available internet data, these models can understand and generate human‐like text in ways previously unachievable. Thus, GPTs are relatively easy to integrate into both new and existing applications as they do not necessarily require the developer to supply their own dataset for the training of the model. In most cases, a simple system prompt is sufficient to achieve the wanted result, significantly reducing developing cost. In the field of virtual patient simulation, it did not only enable educators to improve on existing systems (Gray et al., 2024), but GPTs have also been used to enable conversations between learners and virtual patients directly, thereby unlocking their most transformative potential for improvement. This implementation has elevated the realism of conversations with virtual patients to previously unattained levels, offering a more authentic and engaging learning experience for medical and dental students. Pilot projects utilizing GPT models in virtual patient simulations have been conducted to improve doctors' patient education in anaesthesia (Sardesai et al., 2024), practice history taking of medical students in general medicine (Holderried et al., 2024), as well as in rheumatology (Borg et al., 2024), demonstrating their feasibility for the application for this scenario. However, no such efforts of implementing GPT models in dental virtual patient simulations were evaluated despite the significant opportunities to educate patient encounters and diagnostic interactions. Besides overcoming the lack of real‐life situations in undergraduate dental education, the variety of cases for simulations allows for a broad range of training and reproducibility in teaching endodontic diagnostics. Recently, the adoption of GPT technology in virtual patient simulations also within dental education was proposed (Prinz et al., 2024). However, reliable results on the perception and effectiveness of such approaches in endodontic undergraduate training are still lacking. Thus, this study presents the development and implementation of a GPT‐4 model‐based virtual patient e‐learning tool, specifically designed to enhance endodontic diagnostic skills among undergraduate dental students. The study evaluated students' self‐perception of their endodontic diagnostic abilities and identified any improvements in their self‐perceived skills following practicing.

## MATERIALS AND METHODS

At the beginning of each questionnaire, informed consent was given by the participants. The ethics committee of Westphalia‐Lippe in Münster omitted the necessity of ethical approval for this survey because data were collected anonymously (2025‐230‐f‐N).

### Conceptualization

The initial aim was to build an AI‐driven virtual patient e‐learning platform to enhance students' diagnostic and clinical decision‐making in endodontics, to better prepare them for emergency treatment. This platform should employ a case‐based and observational learning approach, complementing the existing curriculum, by enabling students to practice at any time and from any location. While being designed for individual use primarily, it could be used equally as effective for group sessions providing a safe learning environment. Guidelines such as *Healthcare Simulation Standards of Best Practice™ Simulation Design* (Watts et al., 2021) were included in the planning process. Accordingly, the e‐learning environment had to be effectively structured and to include specific components to facilitate a desired learning outcome. One of these was the pre‐briefing the students undergo initially, which includes an introduction to the setting, instructions on how to use the programme and a guide for a structured approach to endodontic diagnostics. Subsequently, students would engage in the main task—a conversation with a GPT‐4‐powered virtual patient—either by typing or utilizing the voice recognition ability of the programme.

In this simulation, students are challenged to determine the correct diagnosis and recommend suitable treatment options while also addressing any questions the patient may have about their condition or suggested treatment plan. Students are demanded to apply their knowledge on diagnostics, differential therapies and patient education, while simultaneously improving their patient communication skills. To create a realistic training environment, it was thought essential to provide comprehensive patient information for each specific case needed to conclude definitive diagnoses (Levin et al., 2009). This included: general medical history, specific dental history and nature of the pain or discomfort, dental status, radiographs, clinical pictures, findings (responses to clinical tests such as percussion, cold test, palpation of the apical region) and other relevant data. Some of this information, that is, sensitivity tests, needed to be gathered through dialogue to enable a realistic, structured diagnostic approach. Other forms of information, such as clinical images, radiographs or a general medical questionnaire, would not be accessible through the dialogue and, instead, would be available on request. The patient case vignettes had to be precise and extensive to cover all diagnosis‐related information and clinical tests a clinician might ask or perform, so that the AI‐model could give an appropriate answer. To achieve a higher level of realism, each virtual patient was given an individual background and slightly different personalities. Regarding the scope of this pilot study, the focus was on the most common endodontic pathologies only. Hence, eight different patient cases with the following diagnoses based on typical symptoms, clinical and radiographic findings in accordance with the content of the lectures and textbooks provided to the students were created:

- Dentine hypersensitivity,
- Acute reversible pulpitis,
- Acute irreversible pulpitis,
- Acute apical periodontitis,
- Asymptomatic apical periodontitis,
- Cracked tooth syndrome,
- Vertical root fracture,
- Periodontally induced pain.

Considering the level of training and experience of the students, cases and definitions of the pathologies were designed to be explicitly matching the content of the curriculum. Thus, the complexity level was aligned with the expertise of the undergraduate students, which in favour of the learning scope resulted in a rather low level of complexity.

Reconsidering the *Healthcare Simulation Standards of Best Practice™ Simulation Design* (Watts et al., 2021), the session would end once the virtual patient consents to the suggested treatment and is followed by a case‐specific debriefing. There, the students should first receive a brief AI‐generated feedback considering the entire transcript of the previous dialogue. The AI model evaluates the students' proposed diagnosis and treatment against the correct options and provides brief feedback on their patient communication. Then, students would be offered more background information on the present diagnosis as well as a transcript of a conversation between the same patient and an experienced clinician, before being led to the next patient case. In this way, the students could fill theoretical knowledge gaps and compare their procedural approach to the one of their educators. Moreover, if students felt the additional need to discuss their findings with peers or their educators during lectures, an export of their own conversation transcript should be possible.

### Technical implementation

**FIGURE 1:**
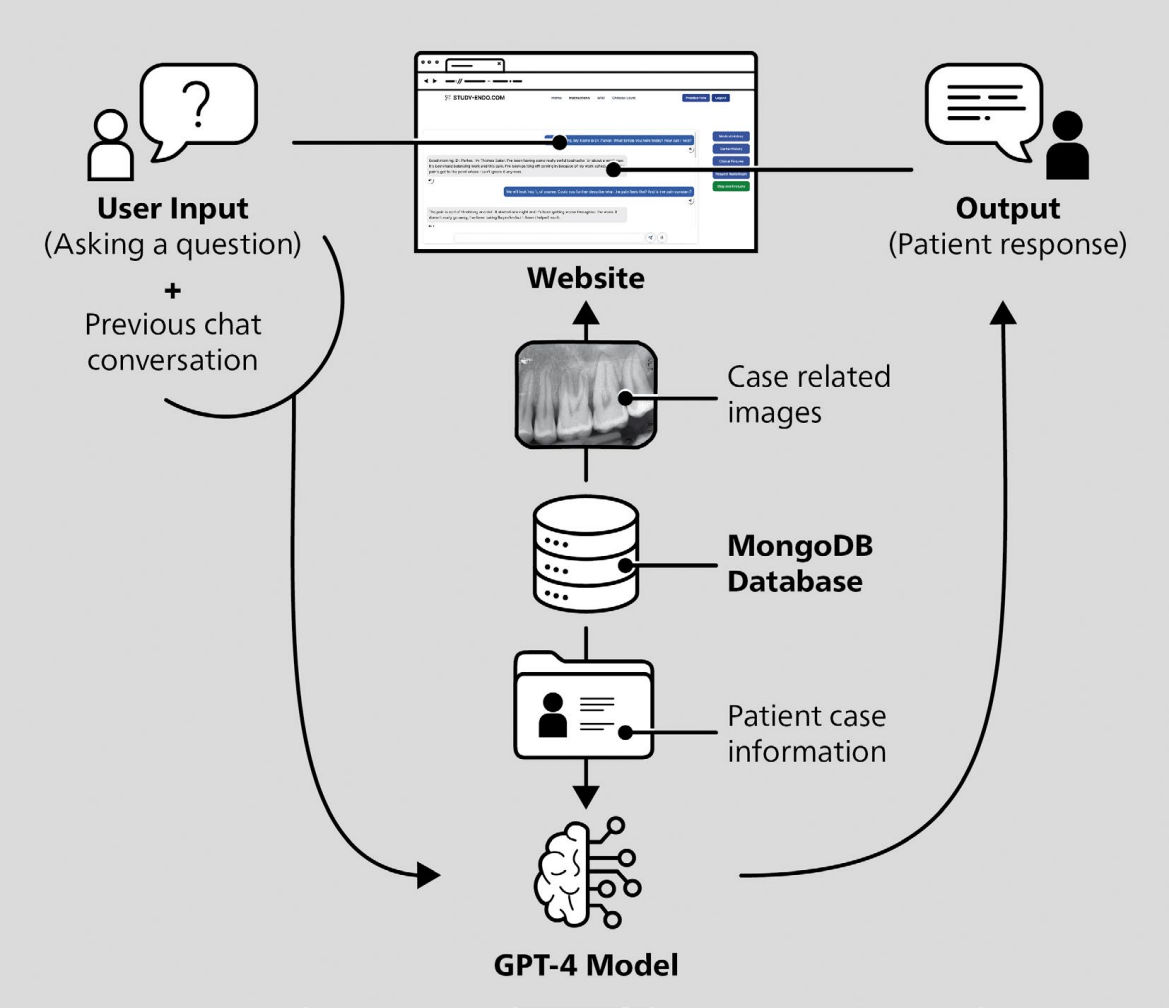
Components and technical implementation of the e‐learning tool's web app.

**FIGURE 2:**
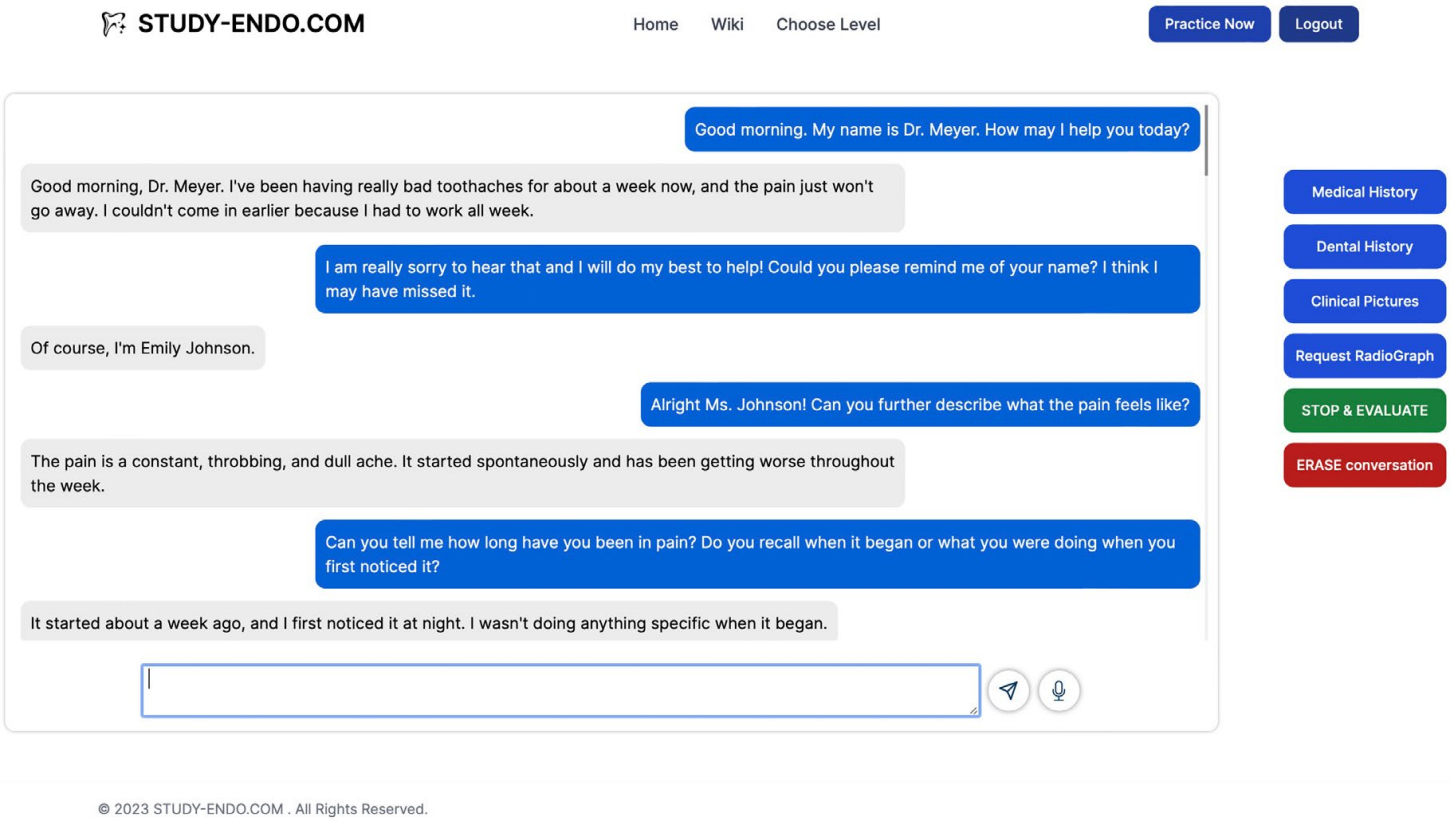
Case specific communication in the web app.

A web app was designed to serve as the e‐learning platform consisting of three main components (Figure 1). First, there is the dedicated website the user can access and interact on. Second, all of the patient case information, including radiographic and clinical images, is stored on a dedicated MongoDB database server (MongoDB Inc., New York, NY, USA). Lastly, to facilitate the interactive nature of the patient cases, the GPT‐4‐turbo model (OpenAI) was employed via its application programming interface (API). Whenever a user poses a question, the third component, the GPT‐4‐turbo model, would not just process the query but the entire preceding dialogue, while also having access to the complete details of the patient case. Additionally, for a higher degree of fidelity, the web app is capable to accept the input of the device‐based speech recognition function and to convert the text output of the GPT‐4 model to audio utilizing the OpenAI text‐to‐speech model, therefore enabling a more realistic conversation between the user and the virtual patient (Figure 2).

### Prompt design

The AI model was instructed within the system prompt to simulate the role of the specific patient with its distinguishing characteristics visiting their dentist, in most cases due to toothache to fit the emergency care objective. The system prompt had to be universal since variables were included as space holders for the different patient cases, stored in the database. Therefore, the prompt and the patient case information had to allow seamless integration of the two, regardless of the specific patient case.

The AI model was instructed explicitly to respond only to questions related to the simulation scenario and to provide answers based solely on the patient case information deposited in the MongoDB database. For questions falling outside the case's scope, the system was programmed to steer the conversation back to the simulation exercise. If case‐related information was unavailable, it was directed to respond with “I am not sure.” The AI model was barred from implementing general information from the world wide web, as this could have resulted in so‐called hallucination. It was also instructed to ask for treatment‐related information from a patient's perspective, once the student concludes a diagnosis and recommends a treatment option. This way the student is required to be able to achieve an informed consent including discussing treatment costs, needed number of appointments and treatment time, as well as risks and alternative treatment options. These instructional prompts were necessary to ensure the model adhered strictly to its role and to minimize the unwanted phenomenon of hallucination, in case a specific user question would not be covered by the patient vignette. To achieve this, the evolution from GPT 3.5 to GPT4, and to GPT4‐turbo, respectively, helped tremendously since the newer models were able to follow their system prompt much more closely.

### Evaluation and perception

After development and consecutive test stages with a selected group of students and teachers (Prinz et al., 2024), a larger rollout served for evaluation of the new e‐learning tool. Fourth‐ and 5th‐year dental school students from the School of Dentistry of the Faculty of Medicine of the University of Münster were asked to participate in the study voluntarily. All of them had, with graduation, experience in clinical dental treatment and theoretical knowledge of endodontic diagnostics. Two questionnaires were disseminated among the participants, the first before using the e‐learning tool (T1), the second after having experienced the tool (T2) (Data S1). The implementation of the AI‐assisted learning tool was evaluated using a Likert scale, the standard procedure for such surveys in the field of medicine (Park et al., 2017; Schlenz, Michel, et al., 2020). The questionnaires were tested in advance on 15 participants who did not take part in the main study for its comprehensibility and average completion time. No personal data was acquired and evaluation by questionnaires was conducted anonymously in an online survey on the cohort level, omitting the necessity of ethical approval.

Prior to introducing the e‐learning tool, a questionnaire (T1) served to evaluate the current self‐perceived state regarding the education in endodontic diagnostics and emergency treatment at the University of Münster. Afterwards, the students were instructed on how to use the program in a short oral introduction and, additionally, referred to the user's and diagnostics guide on the web app. They were granted access to the password protected e‐learning tool thereafter and, after a 3‐month timeframe of working with the programme individually, the students were asked to answer a second questionnaire (T2) which focused on their experience after using the training software (Data S1). Questionnaire 2 terminated with a possibility to freely express thoughts and opinions on the e‐learning tool.

Due to the limited recall rate of the questionnaires on a voluntary basis and the drop‐out rate resulting in limited power for a statistical analysis, the analysis was performed descriptively.

## RESULTS

The web app consisted of the above‐mentioned pre‐briefing introducing the subject and the use of the app, a case selection interface, the chat‐bot interface with accompanying documents on request, and the de‐briefing area. A demonstration of the app is shown in supplementary video (Video S1).

Ninety‐two students from two cohorts participated in the first questionnaire (T1). Among them, 59 students were from 4th year, and 33 from 5th year, respectively. Seventy‐two students finished the second questionnaire (T2) resulting in a drop‐out rate of 21.7%, 20.3% in 4th year and 24.2% in 5th year (Table 1). Students completing the study from 4th year had a mean age of 23.8 years (*n* = 47). 31.9% identified as male and 68.1% as female. 8.5% reported a previous medical or dental qualification. Among 5th‐year students completing the study, 60.0% were female and 40.0% male. They had a mean age of 24.7 years, with 20% reporting medical or dental qualifications. Of the students in 4th year, 85.1% had read the operating guide for the web app, while 78.7% took use of the diagnostics guide. In the 5th year, guides were used in 88% and 68%, respectively.

**TABLE 1:** Participant numbers and drop‐out rates between Questionnaire 1 (T1) and Questionnaire 2 (T2) by year groups.

Cohort	Gender	T1		T2		Dropout
		*n*	%	*n*	%	%
4th year	Male	20	33.9	15	31.9	25.0
	Female	39	66.1	32	68.1	17.9
	Sum	59	100	47	100	20.3
5th year	Male	10	30.3	10	40	0.0
	Female	23	69.7	15	60	34.8
	Sum	33	100	25	100	24.2

In the 4th year, 50.8% of all participants had not encountered exposure to patients suffering from toothache, whereas 42.4% accounted for one or two experiences. Among participants from the 5th year, 48.5% had encountered one or two experiences, and 30.3% up to four encounters with emergency visits, while still 21.2% could not report exposure (Table 2). 38.9% of the participants completed all eight cases (Table 3).

**TABLE 2:** Previous exposure of the participating students to patients suffering from pain of dental origin.

Cohort		0	1 or 2	3 or 4	>5	Sum
4th year	*N*	30	25	0	4	59
	%	50.8	42.4	0.0	6.8	100
5th year	*N*	7	16	10	0	33
	%	21.2	48.5	30.3	0.0	100
Total	*N*	37	41	10	4	92
	%	40.2	44.6	10.9	4.3	100

**TABLE 3:** Number of cases completed by the participants.

		1	2	3	4	5	6	7	8	Sum
4th year	*n*	2	2	5	7	3	4	4	20	47
	%	4.3	4.3	10.6	14.9	6.4	8.5	8.5	42.6	100
5th year	*n*	2	1	5	4	4	1	0	8	25
	%	8.0	4.0	20.0	16.0	16.0	4.0	0.0	32.0	100
Total	*n*	4	3	10	11	7	5	4	28	72
	%	5.6	4.2	13.9	15.3	9,7	6.9	5.6	38.9	100

The results of the initial (T1) and terminatory (T2) questionnaires 1 and 2, respectively, are presented in Tables 4 and 5, respectively.

**TABLE 4:** Results of Questionnaire 1 at Time point 1 (T1): Absolute frequencies and corresponding proportions for each response category based on the applied Likert scale.

Questionnaire No. 1 (T1)												
	Group	Strongly agree (1)		Agree (2)		Neutral (3)		Disagree (4)		Strongly disagree (5)		Sum
		*n*	%	*n*	%	*N*	%	*n*	%	*n*	%	
**1–1 perception of endodontic emergency teaching**												
I have been in contact with emergency patients and pain diagnostics during the clinical treatment course	4th year	2	3.4	6	10.2	10	17.0	21	35.6	20	33.9	59
	5th year	7	21.2	5	15.2	8	24.2	10	30.3	3	9.1	33
	Total	9	9.8	11	12.0	18	19.6	31	33.7	23	25.0	92
Based on the lectures, learning modules, and educational materials available to me so far, I feel well‐prepared to conduct endodontic pain diagnostics	4th year	0	0.0	9	15.3	27	45.8	21	35.6	2	3.4	59
	5th year	1	3.0	6	18.2	16	48.5	10	30.3	0	0.0	33
	Total	1	1.1	15	16.3	43	46.7	31	33.7	2	2.2	92
I would like to have more opportunities to train my diagnostic skills	4th year	43	72.9	15	25.4	0	0.0	1	1.7	0	0.0	59
	5th year	27	81.8	4	12.1	1	3.0	1	3.0	0	0.0	33
	Total	70	76.1	19	20.7	1	1.1	2	2.2	0	0.0	92
I feel sufficiently prepared for the future practice setting in treating emergency patients and do not require additional training beyond the current level available	4th year	0	0.0	1	1.7	7	11.9	18	30.5	33	55.9	59
	5th year	0	0.0	1	3.0	3	9.1	20	60.6	9	27.3	33
	Total	0	0.0	2	2.2	10	10.9	38	41.3	42	45.7	92
**1–2 self‐confidence in an endodontic emergency**												
I feel confident to perform endodontic (pain) diagnostics independently	4th year	0	0.0	5	8.5	18	30.5	21	35.6	15	25.4	59
	5th year	0	0.0	6	18.2	16	48.5	9	27.3	2	6.1	33
	Total	0	0.0	11	12.0	34	37.0	30	32.6	17	18.5	92
I have my own personal and structured approach in diagnostics when carrying out an examination on a patient suffering from dental pain	4th year	0	0.0	8	13.6	20	33.9	17	28.8	14	23.7	59
	5th year	0	0.0	13	39.4	12	36.4	6	18.2	2	6.1	33
	Total	0	0.0	21	22.8	32	34.8	23	23.9	16	17.4	92
I am confident in my ability to carry out an initial diagnosis process independently	4th year	0	0.0	9	15.3	21	35.6	18	30.5	11	18.6	59
	5th year	0	0.0	8	24.2	16	48.5	5	15.2	4	12.1	33
	Total	0	0.0	17	18.5	37	40.2	23	25.0	15	16.3	92
I feel confident explaining endodontic diagnoses and treatment options to patients	4th year	1	1.7	13	22.0	26	44.1	17	28.8	2	3.4	59
	5th year	0	0.0	18	54.5	11	33.3	2	6.1	2	6.1	33
	Total	1	1.1	31	33.7	37	40.2	19	20.7	4	4.3	92

**TABLE 5:** Results of Questionnaire 2 at time point 2 (T2): Absolute frequencies and corresponding proportions for each response category based on the applied Likert scale.

Questionnaire No. 2 (T2)												
	Group	Strongly agree (1)		Agree (2)		Neutral (3)		Disagree (4)		Strongly disagree (5)		Sum
		*n*	%	*n*	%	*n*	%	*n*	%	*n*	%	
**2–1 self‐confidence in an endodontic emergency**												
I feel confident to perform endodontic (pain) diagnostics independently	4th year	4	8.5	25	53.2	16	34.0	2	4.3	0	0.0	47
	5th year	1	4.0	16	64.0	6	24.0	1	4.0	1	4.0	25
	Total	5	6.9	41	56.9	22	30.6	3	4.2	1	1.4	72
I have my own personal and structured approach in diagnostics when carrying out an examination on a patient suffering from dental pain	4th year	9	19.1	26	55.3	10	21.3	2	4.3	0	0.0	47
	5th year	10	40.0	8	32.0	5	20.0	1	4.0	1	4.0	25
	Total	19	26.4	34	47.2	15	20.8	3	4.2	1	1.4	72
I am confident in my ability to carry out an initial diagnosis process independently	4th year	7	14.9	22	46.8	15	31.9	3	6.4	0	0.0	47
	5th year	7	28.0	13	52.0	4	16.0	0	0.0	1	4.0	25
	Total	14	19.4	35	48.6	19	26.4	3	4.2	1	1.4	72
I feel confident explaining endodontic diagnoses and treatment options to patients	4th year	6	12.8	25	53.2	14	29.8	2	4.3	0	0.0	47
	5th year	5	20.0	13	52.0	5	20.0	2	8.0	0	0.0	25
	Total	11	15.3	38	52.8	19	26.4	4	5.6	0	0.0	72
**2–2 perception of AI performance**												
I was able to correctly diagnose all cases and suggest appropriate therapies	4th year	7	14.9	25	53.2	12	25.5	2	4.3	1	2.1	47
	5th year	5	20.0	9	36.0	8	32.0	2	8.0	1	4.0	25
	Total	12	16.7	34	47.2	20	27.8	4	5.6	2	2.8	72
The training scenario was realistic	4th year	20	42.6	17	36.2	8	17.0	2	4.3	0	0.0	47
	5th year	12	48.0	9	36.0	2	8.0	2	8.0	0	0.0	25
	Total	32	44.4	26	36.1	10	13.9	4	5.6	0	0.0	72
It was easy to have a conversation/chat with the AI‐generated patients	4th year	16	34.0	17	36.2	11	23.4	3	6.4	0	0.0	47
	5th year	12	48.0	7	28.0	4	16.0	2	8.0	0	0.0	25
	Total	28	38.9	24	33.3	15	20.8	5	6.9	0	0.0	72
The dialogue seemed linguistically natural and realistic	4th year	15	31.9	17	36.2	9	23.4	6	6.4	0	0.0	47
	5th year	9	36.0	13	52.0	3	12.0	0	8.0	0	0.0	25
	Total	24	33.3	30	41.7	12	16.7	6	8.3	0	0.0	72
It was comfortable to interact with the artificial intelligence	4th year	15	31.9	18	38.3	12	25.5	2	4.3	0	0.0	47
	5th year	13	52.0	9	36.0	1	4.0	2	8.0	0	0.0	25
	Total	28	38.9	27	37.5	13	18.1	4	5.6	0	0.0	72
The AI was able to answer all the questions needed to solve the case	4th year	19	40.4	19	40.4	8	17.0	1	2.1	0	0.0	47
	5th year	14	56.0	7	28.0	4	16.0	0	0.0	0	0.0	25
	Total	33	45.8	26	36.1	12	16.7	1	1.4	0	0.0	72
**2.3 suitability of the AI based learning tool**												
The programme is suitable for improving my diagnostic skills and making me feel more confident in dealing with patients seeking for pain relief	4th year	30	63.8	12	25.5	4	8.5	1	2.1	0	0.0	47
	5th year	16	64.0	6	24.0	1	4.0	1	4.0	1	4.0	25
	Total	46	63.9	18	25.0	5	6.9	2	2.8	1	1.4	72
The programme is not suitable for improving my own diagnostic skills and feeling more confident in dealing with patients suffering from pain	4th year	0	0.0	0	0.0	5	10.6	14	29.8	28	59.6	47
	5th year	0	0.0	2	8.0	3	12.0	5	20.0	15	60.0	25
	Total	0	0.0	2	2.8	8	11.1	19	26.4	43	59.7	72
Using the programme helped me better understand endodontic diagnostics	4th year	22	46.8	19	40.4	5	10.6	1	2.1	0	0.0	47
	5th year	11	44.0	11	44.0	1	4.0	1	4.0	1	4.0	25
	Total	33	45.8	30	41.7	6	8.3	2	2.8	1	1.4	72
I feel better prepared for daily practice after using the programme	4th year	12	25.5	21	44.7	13	27.7	1	2.1	0	0.0	47
	5th year	10	40.0	10	40.0	4	16.0	1	4.0	0	0.0	25
	Total	22	30.6	31	43.1	17	23.6	2	2.8	0	0.0	72
I would like to see more (challenging) cases included in the programme to allow even more training	4th year	33	70.2	12	25.5	2	4.3	0	0.0	0	0.0	47
	5th year	18	72.0	5	20.0	2	8.0	0	0.0	0	0.0	25
	Total	51	70.8	17	23.6	4	5.6	0	0.0	0	0.0	72
In future, the programme should be used for endodontic training and made available to students as a learning tool	4th year	35	74.5	10	21.3	2	4.3	0	0.0	0	0.0	47
	5th year	17	68.0	6	24.0	1	4.0	1	4.0	0	0.0	25
	Total	52	72.2	16	22.2	3	4.2	1	1.4	0	0.0	72
I would recommend this AI‐supported learning method to other students	4th year	36	76.6	9	19.1	2	4.3	0	0.0	0	0.0	47
	5th year	19	76.0	3	12.0	2	8.0	1	4.0	0	0.0	25
	Total	55	76.4	12	16.7	4	5.6	1	1.4	0	0.0	72

	Group	Very good (1)		Good (2)		Average (3)		Poor (4)		Very poor (5)		Sum
		*n*	%	*n*	%	*n*	%	*n*	%	*n*	%	
**2.4 instructions/guide for the programme (only for participants who read the instructions)**												
How do you rate the instructions for using the web app?	4th year	15	37.5	22	55.0	3	7.5	0	0.0	0	0.0	40
	5th year	11	50.0	9	40.9	2	9.1	0	0.0	0	0.0	22
	Total	26	41.9	31	50.0	5	8.1	0	0.0	0	0.0	62
How would you rate the pain diagnosis guide?	4th year	15	40.5	20	54.1	2	5.4	0	0.0	0	0.0	37
	5th year	9	52.9	8	47.1	0	0.0	0	0.0	0	0.0	17
	Total	24	44.4	28	51.9	2	3.7	0	0.0	0	0.0	54

### Initial questionnaire

In the initial questionnaire (T1), perception of endodontic emergency training and self‐confidence in an endodontic emergency situation was evaluated. Values were obtained applying a 5‐point Likert scale [(1) strongly agree; (2) agree; (3) neutral; (4) disagree; (5) strongly disagree]. Neither students of 4th nor 5th year felt to encounter a sufficient number of patients referred for emergency treatment and pain diagnostics. Students from both years had a mainly neutral position on their preparation via lectures and other educational modules and materials on conducting diagnostics. Opinions reported on the desired amount of training in diagnostics were similar in the cohorts, both stating to need more training opportunities. Both cohorts disagreed with the fact of being sufficiently prepared for treatment of dental emergencies without additional training.

Both cohorts reported to mainly not feel confident to perform endodontic diagnostics independently. A difference was observed when asked about having an own and personal approach in diagnostics, on which students from the 4th mostly disagreed and students from the 5th took a neutral or agreeing position. Both cohorts mainly neither agreed nor disagreed on their confidence to be able to find an initial diagnosis correctly and independently. When asked for their confidence in explaining an endodontic diagnosis and treatment options to a patient, students from the 4th year mostly had a neutral position, while students from the 5th year mainly agreed to this statement.

### Terminatory questionnaire

The second questionnaire (T2) asked for self‐confidence in an endodontic emergency situation, the perception of the artificial intelligence (AI) performance, the suitability of the AI‐based learning tool applying the same Likert scale as in questionnaire 1, and an evaluation of the instructions and guide to the web app on a 5‐point Likert scale [(1) very good, (2) good, (3) average, (4) poor, (5) very poor]. Concerning self‐confidence, students from both years mainly agreed on the statement asking for confidence to perform diagnostics independently, having their own structured approach in diagnostics, feeling confident to carry out initial diagnostics independently and being able to explain diagnosis and treatment options to a patient. Students mostly agreed they were able to diagnose the cases and suggest appropriate therapy. They also agreed the training scenario was realistic and conversation with the AI‐generated patients was easy. The same accounted for natural and realistic character of the dialogues, the comfort when interacting with the AI and the ability of the AI to answer all questions necessary to solve the case. Both cohorts strongly agreed that the concept is suitable for improving diagnostic skills and boosting confidence. Both cohorts agreed on an improved understanding of endodontic diagnostics and better preparation for daily practice after using the tool. A strong need was expressed in both cohorts to endure more training with more and more challenging cases. The tool was strongly recommended to be available on demand for endodontic training by both cohorts, paired with the strong recommendation to other students for using the tool irrespective of the cohort. The instructions to the web app and the diagnostic guide were mostly rated good by 4th‐year students and very good by 5th‐year students.

**FIGURE 3:**
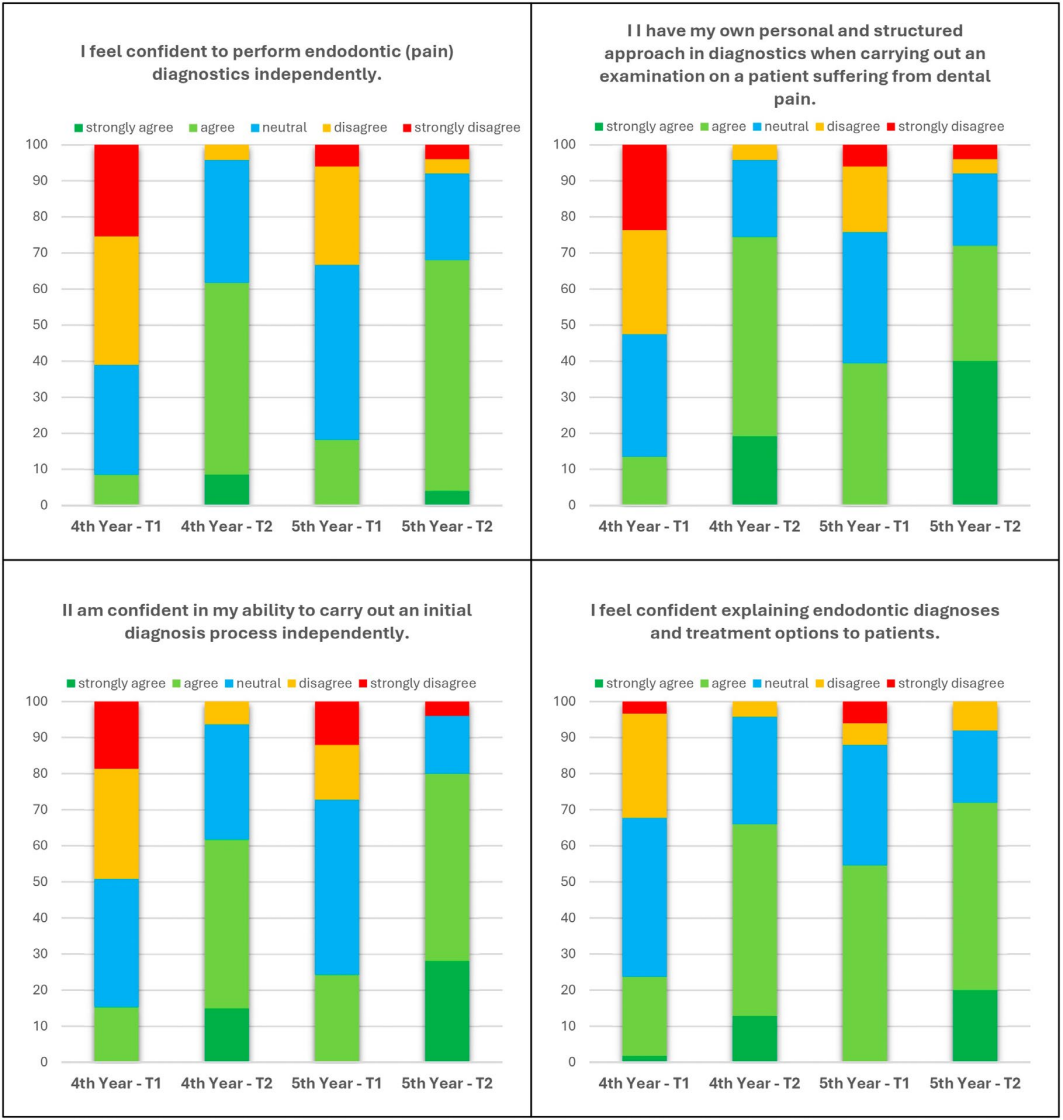
Stacked bar charts displaying the perception of the different cohorts regarding endodontic emergency training before (T1) and after (T2) the use of the e‐learning tool.

Self‐assessment of endodontic diagnostic capabilities, structure of diagnostic approaches, confidence in performing initial diagnostics in an emergency and ability to gain informed consent by explaining diagnosis and therapeutical options were retrieved in duplicate (Table 6). The confidence to perform endodontic diagnostics independently improved both in each cohort (4th‐ and 5th‐year students) and overall after the training on the e‐learning tool. The same accounted for the perception of having an own personal and structured approach in diagnostics when carrying out the examination, the confidence in the personal ability to carry out the initial diagnostics independently and the confidence in explaining endodontic diagnoses and treatment options to patients (Figure 3).

**TABLE 6:** Comparison of self‐assessed items retrieved in duplicate before (T1) and after (T2) the use of the e‐learning tool. Comparison of cohorts in groups A and B, and between totals at T1 and T2.

Question	Group	Time point	Strongly agree (1)	Agree (2)	Neutral (3)	Disagree (4)	Strongly disagree (5)	Sum
I feel confident to perform endodontic (pain) diagnostics independently	4th year	T_1_	0	5	18	21	15	59
	5th year		0	6	16	9	2	33
	Total		0	11	34	30	17	92
	4th year	T_2_	4	25	16	2	0	47
	5th year		1	16	6	1	1	25
	Total		5	41	22	3	1	72
I have my own personal and structured approach in diagnostics when carrying out an examination on a patient suffering from dental pain	4th year	T_1_	0	8	20	17	14	59
	5th year		0	13	12	6	2	33
	Total		0	21	32	23	16	92
	4th year	T_2_	9	26	10	2	0	47
	5th year		10	8	5	1	1	25
	Total		19	34	15	3	1	72
I am confident in my ability to carry out an initial diagnosis process independently	4th year	T_1_	0	9	21	18	11	59
	5th year		0	8	16	5	4	33
	Total		0	17	37	23	15	92
	4th year	T_2_	7	22	15	3	0	47
	5th year		7	13	4	0	1	25
	Total		14	35	19	3	1	72
I feel confident explaining endodontic diagnoses and treatment options to patients	4th year	T_1_	1	13	26	17	2	59
	5th year		0	18	11	2	2	33
	Total		1	31	37	19	4	92
	4th year	T_2_	6	25	14	2	0	47
	5th year		5	13	5	2	0	25
	Total		11	38	19	4	0	72

In the open questions terminatory to questionnaire 2, 11 students expressed positive feedback, with three explicitly stating that they would recommend the programme to their peers. Two students suggested the inclusion of a broader range of disciplines, such as orthodontics. Additionally, two students specifically praised the pre‐briefing wiki. On the technical side, two students reported experiencing system crashes on the website, while another noted that the text input field was initially too small, which was then changed accordingly.

Students also provided suggestions for potential improvements. One student proposed the implementation of a help/info button or an AI assistant to assist users during the virtual patient scenario, particularly when a student is uncertain about how to proceed. Furthermore, two students indicated that the AI feedback could be enhanced, as it only recognizes the correct diagnosis if articulated in a specific manner to the patient, which may not align with typical practices in a real clinical environment. While two students acknowledged that this approach offers a more realistic learning experience for diagnostic procedures compared to traditional textbook study, they pointed out that in actual clinical scenarios, patients exhibit diverse behaviours and not every clinical case is as straightforward as the presented study cases. Lastly, two students expressed a desire for a wider variety of patient cases to be included in the curriculum.

## DISCUSSION

Endodontic diagnostics and emergency patient care are crucial in daily dental practice. Sufficient education demands extensive practice and interaction with real humans. At many universities, students are not involved in clinical emergency care rotations, resulting in a noticeable deficit in self‐efficacy related to emergency patient care (Anderson et al., 2015). This emphasizes the need for additional compensatory educational approaches to close this gap effectively and enhance student preparedness for future clinical encounters. In the present study, an e‐learning web application was used to train undergraduate students in diagnosing dental emergencies presenting with pain of endodontic or periodontal origin (Prinz et al., 2024) and the application was evaluated by students from 4th and 5th year at the University of Münster, Germany. Main findings underline the need for more extensive practical courses on dental emergencies and, furthermore, that practice on the e‐learning tool improved the self‐perceived confidence in the students' diagnostic capabilities.

Efforts were made to improve endodontic teaching by applying computer‐based learning tools (Al‐Madi et al., 2018; Littlefield et al., 2003; Sanchez et al., 2019). With the improvements in digital technologies and advances in artificial intelligence, doors opened for new approaches with more volatile and life‐like encounters. The present study addressed this issue by implementing a virtual patient simulation utilizing GPT‐4 technology. This approach did not only show to enhance the students' self‐efficacy, which is aligned with similar studies (Suárez et al., 2022) but also accustomed them to a routine of diagnosing pain in patients. Utilizing artificial intelligence or simulation and training in dental education allows to practice complex procedures on virtual patients without any risk of harm to real humans (Dave & Patel, 2023). Despite the flexibility accompanying e‐learning, simulations present a safe environment for students, allowing them to adopt their theoretical skills into practice.

Consequently, after utilizing the programme, students reported adopting a more standardized and structured approach to clinical diagnostics. This improvement indicates that virtual training tools could effectively prepare students for initial patient interactions in emergency care settings or serve as a vital substitute for a lack of clinical patient encounters. Due to this new approach, comparable studies are sparse. Still, comparable conclusions were drawn from less advanced simulations of patient encounters (Littlefield et al., 2003; Schittek Janda et al., 2004). However, this study focused solely on the students' perception of their own capabilities. Hence, a limitation could be a mismatch with an actual and objectively measured acquisition of skills. Future research should aim to evaluate skill development to provide a more comprehensive understanding of the effectiveness of these novel virtual simulations compared to traditional VP approaches that have already proved to be effective. Other limitations of the present study are the limited number of participants and the voluntary participation of students, which may introduce a selection bias as more motivated and engaged students are more likely to participate. The drop‐out rate from the first to the second questionnaire emphasizes this thesis. Some participants might have felt not to have enough time to finish a sufficient number of cases as the cases were quite time‐consuming, adding up to approximately 3–4 hours of practice on a voluntary and non‐curricular basis. Although many invested a few minutes to fill in the first questionnaire, they apparently were not willing to invest their time for this project and hence did not fill in the second questionnaire. Those who finished both questionnaires evaluated the accessibility of the tool both in the Likert scales and in free texts positively, making accessibility an unlikely reason for the drop‐out. The final participants might have felt a strong interest in improving their skills—a prerequisite that could not prove right for every dental student—and consequently had a self‐interest in successful participation.

Despite these limitations, this pilot study inherits several strengths. Unlike earlier studies (Suárez et al., 2022), the simulation integrated comprehensive diagnostic findings, including clinical images and radiographs, providing students with nearly all the information available in real‐life scenarios to support their diagnostic conclusions in the virtual simulation. Utilizing the GPT‐4 Model to power the conversation, has been beneficial as well concerning the low set‐up cost and effort compared to the approach by Kim et al. and is more scalable and easier to include new patient cases then approaches using dialogflow (Suárez et al., 2022). Moreover, prioritizing to optimize the learning experience by guiding students through a well‐structured programme that included pre‐briefing, supportive materials and debriefing leads to an integration of different aspects of endodontic diagnostics with likely enhancing the learning experience and success.

Apart from the application of AI and GPT‐Technology as a virtual patient, there might be other potential roles in dental education. With the right training data, the creation of an AI dental educator that can patiently answer all the questions of a student might be a conceivable perspective. In the context of the present study, this would be a great way to improve the feedback following the practice sessions on a virtual patient and furthermore a possibility to use resources inside a dental school more efficiently. Moreover, this technology could also be explored as a dynamic educational tool for diagnostic assistance. Besides other applications of AI, the field of AI‐driven virtual patient simulation offers a variety of opportunities for further developments. Improving the simulation through reduced latency in voice recognition and text‐to‐speech functions is a potential area for development. With the rapid advancements in AI technology, the capabilities of existing LLMs are expected to improve significantly while costs decrease. Already, it is appearing to be feasible to consider replacing the OpenAI GPT‐4 model with more cost‐effective or even free open‐source alternatives, which it was not when we set out to develop this e‐learning tool. This substitution could substantially lower costs, facilitating an expansion into a publicly accessible platform for all interested universities and students, where universities could share patient cases of their own to enhance the overall variety of cases. Another viable approach would be the repurposing of older virtual patient cases, as previously suggested (Antoniou et al., 2014). Advances could even lead to the creation of a virtual reality environments featuring fully examinable virtual patients (Joda et al., 2018; Li et al., 2021). In the short term, integrating these virtual experiences with interactive analogue benchtop simulations through augmented reality (Joda et al., 2019) could provide a more engaging training environment in preclinical courses as a part of the dental curriculum.

## CONCLUSION

Within the limitations of self‐assessment of diagnostic skills, the present study suggests that current AI technology allows to run virtual patient encounters on a sufficient level to improve endodontic diagnostic skills of undergraduate students, when the details of each case scenario are predefined. Not only did the e‐learning tool improve the perception of diagnostic skills of undergraduate students but also presented an environment where students were able to test their skills without potential harm to real‐life patients. AI‐based learning programmes allowing for complex conversational patient encounters could be a possibility to improve diagnostic and interactive skills, and implementation into endodontic curricula could be considered.

## AUTHOR CONTRIBUTIONS

**Marian Prinz:** conceptualization, methodology, software, investigation, writing—original draft, visualization. **Edgar Schäfer:** validation, resources, writing—review & editing. **Sebastian Bürklein:** conceptualization, methodology, validation formal analysis, investigation, data curation, writing—review & editing. **David Donnermeyer:** conceptualization, methodology, investigation, data curation, writing—original draft, visualization, supervision, project administration.

## CONFLICT OF INTEREST STATEMENT

The authors declare no conflict of interest.

## ETHICS STATEMENT

The ethics committee of Westphalia‐Lippe in Münster omitted the necessity of an ethical approval for this survey because data was collected anonymously (2025‐230‐f‐N).

## Supporting information


Data S1.

Video S1.

## Data Availability

Data available on request to the authors.
