# Supplementary material for: Endodontic diagnostics training in undergraduate dental education: An observational pilot study on AI‐driven virtual patient e‐learning
Source: Int Endod J. 2025 Jul 22;59(6):1145–59. doi: 10.1111/iej.14277 (PMC13158537; doi:10.1111/iej.14277)
Supplement: Supplementary file 1 — Data S1. [file IEJ-59-1145-s002.pdf]

Questionnaire No. 1

**Questions**	**answers**<br>**(only one to be selected)**
1. age	18-99
2. gender identity	female / male / other / do not disclose
3. Which clinical course are you currently attending?	4th Year / 5th Year
4. Have you received prior education in a medical/dental context?	Yes / No
5. I performed diagnostics on this amount of emergency patients in clinical courses:	0 / 1-2 / 3-4 / >5
6. I have been in contact with emergency patients and pain diagnostics during the clinical treatment course.	1-Strongly Agree<br>2-Agree<br>3-Neutral<br>4-Disagree<br>5-Strongly Disagree
7. Based on the lectures, learning modules, and educational materials available to me so far, I feel well-prepared to conduct endodontic pain diagnostics.	
8. I would like to have more opportunities to train my diagnostic skills.	
9. I feel sufficiently prepared for the future practice setting in treating emergency patients and do not require additional training beyond the current level available.	
10. I feel confident to perform endodontic (pain) diagnostics independently.	1-Strongly Agree<br>2-Agree<br>3-Neutral<br>4-Disagree<br>5-Strongly Disagree
11. I have my own personal and structured approach in diagnostics when carrying out an examination on a patient suffering from dental pain.	
12. I am confident in my ability to carry out an initial diagnosis process independently.	
13. I feel confident explaining endodontic diagnoses and treatment options to patients.	

Questionnaire No. 2

Questions	answers (only one to be selected)
1. age	18-99
2. gender identity	female / male / other / do not disclose
3. Which clinical course are you currently attending?	4th Year / 5th Year
4. Have you received prior education in a medical/dental context?	Yes / No
5. Number of patient cases I have worked on in the program:	0-8
6. I have read the user guide for the program.	Yes / No
7. I have read the clinical pain diagnosis guide:	Yes / No
8. I feel confident to perform endodontic (pain) diagnostics independently.	1-Strongly Agree<br>2-Agree<br>3-Neutral<br>4-Disagree<br>5-Strongly Disagree
9. I have my own personal and structured approach in diagnostics when carrying out an examination on a patient suffering from dental pain.	
10. I am confident in my ability to carry out an initial diagnosis process independently.	
11. I feel confident explaining endodontic diagnoses and treatment options to patients.	
12. I was able to correctly diagnose all cases and suggest appropriate therapies.	1-Strongly Agree<br>2-Agree<br>3-Neutral<br>4-Disagree<br>5-Strongly Disagree
13. The training scenario was realistic.	
14. It was easy to have a conversation/chat with the AI-generated patients.	

15. The dialogue seemed linguistically natural and realistic.	
16. It was comfortable to interact with artificial intelligence.	
17. The AI was able to answer all the questions needed to solve the case.	
18. The programme is suitable for improving my diagnostic skills and making me feel more confident in dealing with patients seeking for pain relief.	1-Strongly Agree<br>2-Agree<br>3-Neutral<br>4-Disagree<br>5-Strongly Disagree
19. The programme is not suitable for improving my own diagnostic skills and feeling more confident in dealing with patients suffering from pain.	
20. Using the programme helped me better understand endodontic diagnostics.	
21. I feel better prepared for daily practice after using the programme.	
22. I would like to see more (challenging) cases included in the programme to allow even more training.	
23. In future, the programme should be used for endodontic training and made available to students as a learning tool.	
24. I would recommend this AI-supported learning method to other students.	
25. How do you rate the instructions for using the web app?	1-Very good<br>2-Good<br>3-Average<br>4-Poor<br>5-Very poor
26. How would you rate the pain diagnosis guide?	
